# The sky's the limit: improving satellite imagery data literacy to address non-communicable diseases

**DOI:** 10.1093/inthealth/ihae076

**Published:** 2025-01-09

**Authors:** M Courtney Hughes, Elizabeth J Folkmann

**Affiliations:** Department of Public Health, Northern Illinois University, 209 Wirtz Hall, DeKalb, IL 60115, USA; Max Nader Center for Rehabilitation Technologies and Outcome Research, Shirley Ryan Ability Lab, 355 East Erie Street, Chicago, IL 60611, USA

**Keywords:** artificial intelligence, data literacy, developing countries, machine learning, non-communicable diseases, satellite imagery

## Abstract

Low-and middle-income countries experience 77% of the world's premature deaths caused by non-communicable diseases, and their underlying health determinant data are often scarce and inaccurate. Improving satellite imagery data literacy worldwide is an integral step toward using the vast amount of publicly available data collected via satellites, such as air pollution, green space and light at night—all determinants of non-communicable diseases. Existing machine learning–based algorithms enable automated analysis of satellite imagery data, but health officials and scientists must know where to find and how to apply these algorithms to measure risk and target interventions.

The World Health Organization Sustainable Development Goal Target 3.4 aims to reduce premature non-communicable disease (NCD) mortality by one-third by 2030.^1^ NCDs account for 74% of global deaths annually, of which 77% occur within low- and middle-income countries (LMICs). Furthermore, there are 17 million annual premature NCD deaths (before age 70), of which 86% are within LMICs.^2^ One challenge to reducing the global NCD burden is the scarcity of health determinant data available in LMICs to identify risk factors, allocate resources and monitor progress. Satellite imagery data can help fill this gap. In this article, we highlight the importance of improving satellite imagery data literacy, or knowledge and competency, in using satellite imagery data to address NCDs.

Traditional (non-satellite-derived) environmental metrics derived from surveys (e.g. light at night or greenspace surveys) and ground monitoring stations (e.g. air pollution monitoring stations) have limitations. Survey data can be subjective, resource intensive and limited to one or just a few points in time. Ground monitoring station data are limited to the specific geographic area near the station—most often a developed urban area in a high-income country—leading to challenges in extrapolating the data to other locations. Satellite data overcome these limitations with their broad geographic coverage, consistent data quality, open accessibility and remote sensing capability.

The National Aeronautics and Space Administration (NASA) and the European Space Agency (ESA) enable scientists to use satellite data, also called earth observation (EO) data, in their research by offering freely available resources and instruction. For example, through websites and extensive online tutorials, these agencies guide scientists in determining the optimal scale of data given the scientist's computer processing power and their research questions. The agencies’ websites also provide information about optimizing the four resolutions of satellite imagery data—spatial, temporal, radiometric and spectral. Additionally, NASA and the ESA provide validated standard algorithms (e.g. the normalized difference vegetation index for measuring green space) to facilitate uniform comparisons across regions and time.

Despite the emergence of machine learning—using data and algorithms to create autonomous or semi-autonomous learning—there has been little use of this form of artificial intelligence in processing satellite imagery data to address NCDs.^3^ Scepanovic et al.^4^ addressed this gap by creating MedSat, a user-friendly dataset with sociodemographic, environmental, seasonal satellite image band and prescription outcome features designed for studying the relationship between the environment and population health in specific areas of England in 2019 and 2020. MedSat enables using repeat methodology to create more robust datasets inclusive of satellite-derived data.

Improving satellite imagery data literacy is essential to allow health officials and scientists to use satellite imagery to help address NCDs. First, it provides individuals with knowledge about where to find satellite imagery data, tutorials and tools. Second, improving literacy in this area includes teaching health officials and scientists skills to interpret satellite data accurately. For example, satellite imagery users for health purposes can learn to identify and analyse various features and patterns within the imagery, such as land cover changes, vegetation health and urban sprawl. Third, improving satellite imagery data literacy facilitates communication and collaboration among interested individuals and fosters a global community that can share knowledge and support further innovation.

LMICs are likely to experience considerable advantages from improving satellite imagery data literacy as it relates to NCDs. Environmental risk factor surveillance is lacking, and that which exists often has major inaccuracies. For example, a recent systematic review on air pollution and the risk for asthma and respiratory diseases in LMICs revealed a gap in such data, finding just three studies published since 1946 on this topic.^5^ Using measures derived from satellite imagery and previously developed indices arms health officials and researchers focused on LMICs with the necessary information to track risks over time and benchmark against other LMICs and higher-income countries.

As with other sophisticated data sources, when they first become available, experts who are knowledgeable and skilled in their use benefit themselves and their organizations with the information and insights derived from the data. The democratization of satellite imagery data literacy will require making this data and the tools used to process it accessible to a much broader population. To address NCDs, health officials and researchers must know where to obtain satellite imagery data and how to use the data to answer critical epidemiological questions such as ‘In what geographical areas is NCD risk highest?’ and ‘Where has NCD risk changed over time?’. While answers may already exist to some of these questions, adding satellite imagery data will lead to more accurate answers, especially for geographic areas such as LMICs that have not been examined as much as their counterparts.

As technology advances and innovations to obtain and analyse satellite imagery data emerge, literacy in this area must continue evolving. NCDs represent a global health crisis with profound harm to society. Satellite imagery holds tremendous potential to address the multifaceted challenges posed by NCDs. Improving satellite imagery data literacy will facilitate global public health research that can better navigate complex environmental health data obtained from space and make informed decisions that lead to decreasing the prevalence of NCDs in our world. Realizing this potential requires a concerted effort from governments and scientists across the globe to leverage this technology with better education to build a healthier future for all.

## Data Availability

This is a commentary, and data is not available.
